# Computational modelling of micropolar blood-based magnetised hybrid nanofluid flow over a porous curved surface in the presence of artificial bacteria

**DOI:** 10.3389/fchem.2024.1397066

**Published:** 2024-06-05

**Authors:** Wejdan Deebani, Zahir Shah, Muhammad Rooman, Naeem Ullah Khan, Narcisa Vrinceanu, Meshal Shutaywi

**Affiliations:** ^1^ Department of Mathematics, College of Science and Arts, King Abdulaziz University, Rabigh, Saudi Arabia; ^2^ Department of Mathematical Sciences, University of Lakki Marwat, Lakki Marwat, Pakistan; ^3^ Faculty of Engineering, Department of Industrial Machines and Equipment, “Lucian Blaga” University of Sibiu, Sibiu, Romania

**Keywords:** hybrid nanofluid, Eyring–Powell fluid model, micropolar blood-based flow, porous medium, Joule heating, curved boundary

## Abstract

This work provides a brief comparative analysis of the influence of heat creation on micropolar blood-based unsteady magnetised hybrid nanofluid flow over a curved surface. The Powell–Eyring fluid model was applied for modelling purposes, and this work accounted for the impacts of both viscous dissipation and Joule heating. By investigating the behaviours of Ag and TiO_2_ nanoparticles dispersed in blood, we aimed to understand the intricate phenomenon of hybridisation. A mathematical framework was created in accordance with the fundamental flow assumptions to build the model. Then, the model was made dimensionless using similarity transformations. The problem of a dimensionless system was then effectively addressed using the homotopy analysis technique. A cylindrical surface was used to calculate the flow quantities, and the outcomes were visualised using graphs and tables. Additionally, a study was conducted to evaluate skin friction and heat transfer in relation to blood flow dynamics; heat transmission was enhanced to raise the Biot number values. According to the findings of this study, increasing the values of the unstable parameters results in increase of the blood velocity profile.

## 1 Introduction

Micropolar fluids are a type of polar fluid that have microscopic features located in the non-symmetric stress tensor. The presence of inflexible, spherical, or randomly oriented particles is a characteristic of a micropolar fluid. These particles show distinct microrotations and spins when suspended in a viscous liquid. These fluids are capable of a wide variety of microscale actions. Micropolar flow can be observed in many physical phenomena, such as blood flow, bubbling liquids, and liquid crystals. The most recent work on micropolar fluids by Xu and Pop (2014) combines advancements in nanofluids (NFs) and bioconvection. Aziz et al. (2012) theorised the boundary layer flow of the NF, including bioconvection. Agarwal et al. (1989) examined micropolar fluid flow across a stretched sheet using finite-element method for flow and heat transport solutions. Studies were conducted on the steady boundary layer flow across impermeable and permeable sheets in the presence of micropolar flow by Hassanien and Gorla (1990). Eringen (1966a) originally proposed the notion of micropolar fluids, and Abd El-Aziz (2013) studied the manner in which rotational dynamics behaved in micropolar fluids through individual particle motions. Spin inertia plays a role in maintaining the rotational momentum and stress in a body. The stagnant axisymmetric flow of a micropolar NF inside a rotating cylinder was studied by Nadeem et al. (2012). Balaram and Sastri (1973 explored free convection flow in a vertical parallel plate using a micropolar fluid. The two-dimensional flow at the asymmetric stagnation point was studied by Lok et al. (2003).

The goal of producing NF composites is to enhance the characteristics that distinguish nanoparticles, such as thermal conductivity. Framing can be used to produce NFs that are useful for increasing the volume of fluids so as to absorb thermal energy and enhance the rheological aspects. Accordingly, the enhanced thermal energy and rheological characteristic transfer in liquids render NF composites indispensable. Two nanoparticles can be combined to form a hybrid nanofluid (HNF); these nanoparticles, which are made of metals, oxides, or carbides, normally have sizes in the range of 1–100 nm. HNFs are significant in medicine, solar energy, and nuclear applications because they improve heat conductivity. Waini et al. (2019) investigated a HNF with variable volume fraction for copper nanoparticles and a fixed volume fraction of 0.1 for alumina nanoparticles flowing over a moving permeable surface. Sulochana et al. (2020) studied HNFs beyond the wedge and cone and found evidence of nonlinear radiation. Ashwinkumar et al. (2021) employed nonlinear thermal radiation to explore the flow of a $CuO-Al_{2}O_{3}$ water HNF through a vertical cone and plate; they investigated the features of HNF flow while considering two altered geometries. Samrat et al. (2021) investigated the influence of a stretched surface on heat transmission in dusty and dusty HNF flows. Acharya (2021) devised a quasi-linearisation modelling technique to study the hydrothermal properties of HNFs employed on tilted spinning discs. Sarwar and Hussain (2021) examined human blood flow under the assumption of stenosis. Using the Cattaneo–Christov heat flux model and by taking the heat generating effects into consideration, Garia et al. (2021) studied the flow behaviours of HNFs over two different geometries; they discovered that the Cattaneo–Christov model exhibited superior accuracy for predicting heat transport behaviours compared to the Fourier heat flow model. Eringen (1966b) introduced the micropolar fluid model as a well-founded and major generalisation of the classical Navier–Stokes model by accounting for the many additional phenomena that occur in theory and applications. Hassanien and Gorla (1990) analysed heat transfer from a stretched sheet to a micropolar fluid and solved the model mathematically. Subhani and Nadeem (2019) deliberated the time-dependent micropolar magnetohydrodynamic (MHD) fluid flow in a permeable medium for a two-dimensional plane.

The term “fluid” under the influence of magnetic and electromagnetic forces is referred to as “magnetohydrodynamic” (MHD). Solar panels, polymer manufacturing, and highly conductive boilers are a few examples of applications that utilise MHD behaviours. Various studies have been conducted in this field because scientists aim to maintain the NFs under the influence of electromagnetic forces. The micro liquid squeezing flow in a medium under the effect of a magnetic field was investigated by Ghadikolaei et al. (2017). Ullah et al. (2017) examined the non-Newtonian fluid flow numerically over an enlarging sheet in the presence of a magnetic field using the Keller box method. Gul et al. (2015) studied the heat transport of a ferrofluid using a vertical tube in a magnetic field. Saqib et al. (2018) examined the flow of carbon-nanotube-based NFs in a channel under natural convection constraints; their results indicate that greater heat transfer improvement is attained when using lower volume fractions in comparison with the base fluid. Ma et al. (2019) conducted a numerical investigation on the MHD natural convection of NFs in a U-shaped baffled enclosure; they noted that the length of the baffle considerably impacts the flow and temperature patterns. It was also shown that heat transmission increases as the Hartman number increases at low Rayleigh numbers but decreases at large Rayleigh numbers. Khan et al. (2020) examined how the shapes of nanoparticles affect the features of peristaltic flow in MHD NFs within an asymmetric channel; their findings reveal that platelet-shaped particles exhibit higher heat transmission rates than brick or cylinder forms. Ghalambaz et al. (2019) studied the phenomenon of spontaneous convection in a confined space filled with a mixture of copper and aluminium oxide nanoparticles, which are known as Cu–Al_2_O_3_ HNFs, where enclosure was separated by a flexible membrane; they discovered that increasing the quantity of solid particles in the fluid improved heat transfer. On the other hand, the flexibility of the membrane caused a delay in the circulation of the NF. Equations were also derived to calculate the Nusselt number. Aman et al. (2018) provided precise solutions for the flow of a hybrid Casson NF in a porous medium by taking into account the MHD effects; this study demonstrated that the Casson and magnetic factors had substantial impacts on the velocity, nanoparticle concentration, and temperature. Das et al. (2017) studied the entropy produced for the flow of Cu–Al_2_O_3_ HNFs in an absorbent channel under MHD stimulus; their results indicate that there is a decrease in entropy production with increase in the volume fraction; the entropy generation number exhibited a positive correlation with the Hartman and Brinkman numbers. Anantha Kumar et al. (2020a) explored micropolar fluid flow with viscous dissipation effects over a thin stretching surface using a modified Fourier heat flux model for the analysis; they discovered that the temperature profiles decreased as the levels of viscous dissipation and micropolar parameters increased. Ramadevi et al. (2020) inspected the mixed convection flows of micropolar fluids by employing a modified Fourier heat flux model; their findings indicate that the velocity profiles decrease and the temperature and concentration profiles increase when considering the magnetic and viscous dissipation factors. Dawar et al. (2020) examined the chemically reactive MHD flows of micropolar NFs by considering the impacts of velocity slips and changing heat generation/absorption; they noted positive correlations between the temperature profiles and reaction rate parameters as well as negative correlations with heat absorption effects. Scientists and engineers are fascinated by the wide range of industrial applications of stretching sheets because of their heat transfer and boundary layer flow; these applications are often diverse and include hot rolling, gas blowing, metal spinning, wire drawing, polymer sheet extruding, and liquid composite moulding. Sakiadis (1961) described the results of constant speed over a solid boundary wall. Tsou et al. (1967) investigated the properties of heat transfer on a stretching sheet. Crane (1970) examined an analytical solution for the viscous fluid flow induced by a linearly stretched surface. To account for suction or blowing, Gupta and Gupta (1977) conducted an analysis of the effects of linear velocity across a stretchy sheet. Grubka and Bobba (1985) considered the linear velocity with changing temperature distribution while analysing the heat transfer characteristics. Magyari and Keller (1999) investigated exponential flow velocity and heat transmission with thermal dispersion effects over a stretching surface. Elbashbeshy (2001) extended the work by Keller and Magyari to determine how wind and suction affect a surface. Other relevant research works can be explored through references (Shah et al., 2024a; Grigore et al., 2017; Hasegan et al., 2019; Boicean et al., 2023; Shah et al., 2024b), from which readers may explore further into the subject matter to gain an inclusive understanding of the research landscape.

Scholars and mathematicians have been increasingly interested in studying fluid dynamics over curved surfaces because of their importance in technology and engineering. This phenomenon has significant consequences in several engineering disciplines, including fabrication of polymer sheets, rubbers, melt-spinning, paper making, and fibreglass. Micropolar fluid flow over a curved surface is a complex phenomenon that occurs in many scientific and practical applications. Scholars often explore complex flow patterns using mathematical models and numerical simulations to develop innovative methods for regulating and enhancing fluid flows in a range of settings. Alblawi et al. (2019) numerically investigated a curved surface that had been stretched exponentially. Ahmad and Khan (2019a) studied the effects of fluid flow using magneto-nanomaterials on a porous curved surface; the authors also conducted a computer study of the MHD movement of a Sisko nanomaterial fluid over a curved surface Ahmad and Khan, (2019b). Sheikholeslami et al. (2020) developed a mathematical model for MHD-based fluid flow of a nanomaterial over an inclined surface. Sajid et al. (2010) studied the flow of a micropolar fluid over a curved stretching surface. The continuous, incompressible flow of micropolar fluid across an exponentially curved surface was assessed by Shi et al. (2021) using a Keller box approach. Okechi et al. (2017) established the flow across a curved surface by accounting for the velocity and exponential similarity factors. Abbas et al. (2022) examined the time-dependent flow properties of a magnetised micropolar fluid adjacent to a curved surface. Anantha Kumar et al. (2020b) examined the Casson fluid issue in the context of heat radiation-influenced exponentially stretched curved surfaces. Other simulations and investigations of entropy analyses with applications may be seen in Ghachem et al. (2018), Elgazery et al. (2022), Rooman et al. (2022), and Maatki (2023).

### 1.1 Objective

The aim of this work was to computationally model and numerically analyse the behaviours of an unsteady magnetised micropolar blood-based HNF containing gold and copper nanoparticles as it flows over a curved surface. The Powell–Eyring fluid model was used in this study, and consideration was given to the viscous dissipation and Joule heating processes. The model objective was to contrast the performances of the HNF models. By investigating the behaviours of Ag and TiO_2_ nanoparticles dispersed in blood, our aim was to understand the intricate phenomenon of hybridisation. Artificial bacteria swim in an interstitial nanoliquid that is heated by Joule heating and variable thermal conductivity within a biotic cell. The effects of velocity slip and thermal jump were considered while analysing the curved surface. The basic flow assumptions were considered in the development of a mathematical framework. The numerical technique used to design the model verified previous analyses, demonstrating good agreement for a specific circumstance. The investigations delve into the impacts of various parameters, such as the Prandtl number, volume fraction of the nanoparticles, and blood flow parameters. The outcomes were meticulously elucidated through graphical representations and tabular summaries.

### 1.2 Novelty of the investigation

This study is unique and has a novel methodology because of the following:

• The integration of the different elements involved in the flow phenomenon has multiple implications for biomedical and technical applications.
• The use of a blood-based NF containing gold nanoparticles and blood-based HNF incorporation in combination with Ag particles introduces novel characteristics to a real-life physiological fluid.
• The curved surface consideration is useful for imitating medical devices with spinning components.
• The role of dissipative heat in conjunction with particle concentration is specifically attractive for the blood flow phenomenon, thereby improving the heat transmission qualities.
• We performed comparisons between the behaviours of an unsteady magnetised micropolar blood-based NF containing gold nanoparticles and a blood-based HNF incorporating both gold and copper nanoparticles.
• Gold nanoparticles (GNPs) are able to treat and kill cancerous tumours because of their large atomic numbers, which generate heat and aid in the treatment of the tumour. GNPs possess several other attributes that are critical for the treatment of cancer; even though they are small, they have the ability to penetrate deep within the body.
• Significantly, the findings of this research bear relevance to a broad spectrum of biomedical applications.

## 2 Model description

### 2.1 Formal model and geometry

We considered a two-dimensional unsteady boundary layer bioconvection flow of the Eyring–Powell micropolar blood-based magnetised HNF (containing Ag and TiO_2_ nanoparticles) over a porous curved stretching surface. The curvilinear coordinate system was chosen, where r,s are the radial components, s is the length of the arc, and stretching velocity in the s direction is $u=U_{w}=\frac{as}{1-ct}$ , and r is perpendicular to the tangent. The variable magnetic field intensity $B\left(t\right)$ operates normal to the surface. To precisely describe the dynamics of bacterial density $\rho_{n}$ and nutrient concentration n , the reaction–diffusion equations are utilised.

• Unsteady, incompressible, and 2D boundary layer bioconvection flows of the Eyring–Powell micropolar blood-based magnetised hybrid NF (containing Ag and TiO_2_ nanoparticles) over a porous curved stretching surface are considered.
• The curvilinear coordinate system was chosen, where r,s are the radial components and s is the length of the arc.
• The velocity in the s direction is $u=U_{w}=\frac{as}{1-ct}$ , and r is perpendicular to the tangent.
• A varying magnetic field acts normal to the surface.
• A variable heat transfer is assumed.
• To describe the dynamics of bacterial density $\rho_{n}$ and nutrient concentration n , the reaction-diffusion equations are utilised.

**FIGURE 1:**
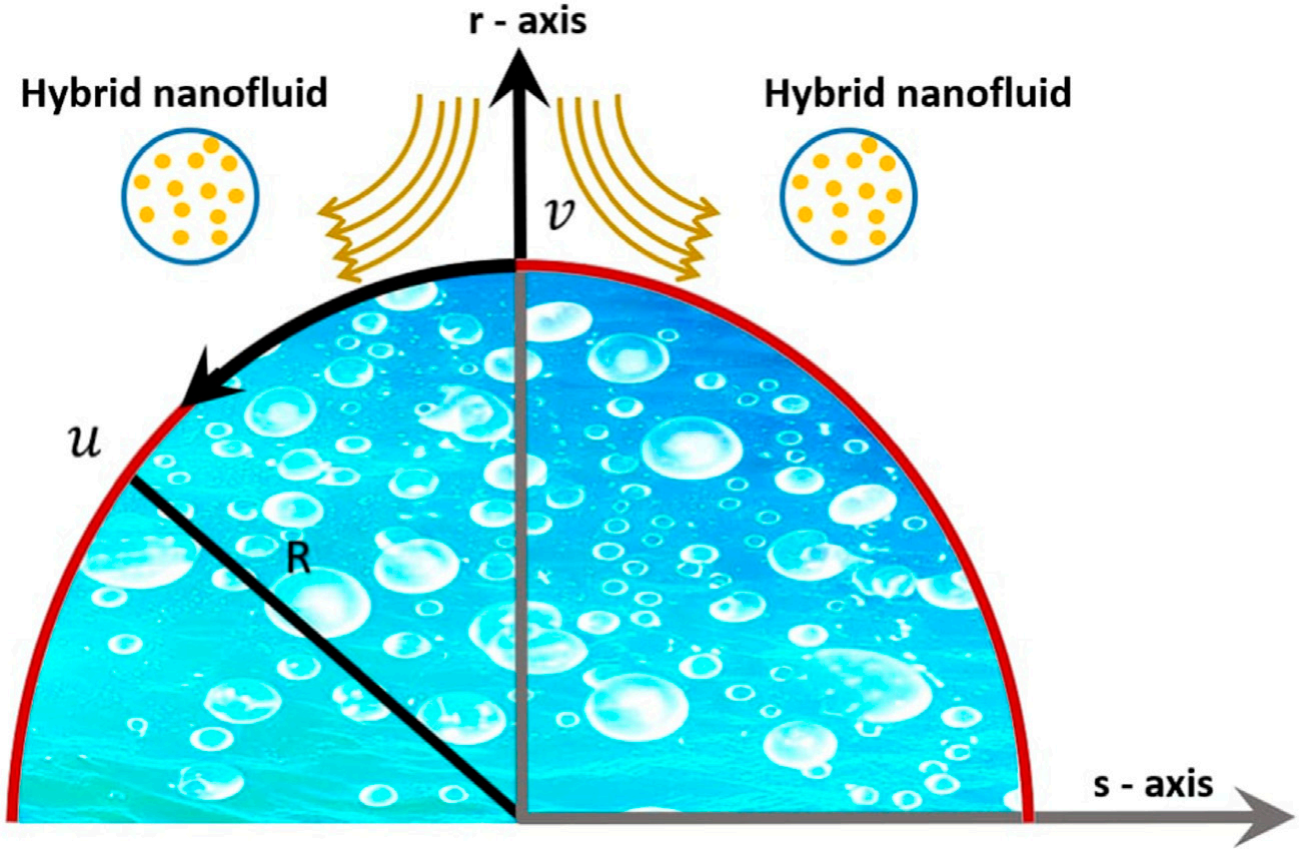
Fluid flow configuration and coordinate system.

Figure 1 illustrates the coordinate system, velocity field, and relevant elements of the flow problem geometry.

### 2.2 Mathematical formulation and basic equations

The constitutive equations characterising micropolar behaviour are constructed in vectorial form using new kinematic properties like the gyration tensor and micro-inertia moment tensor, in addition to the body moments, stress moments, and micro-stress. To take into consideration the magnetic effects, these equations are further enhanced, as follows (Ashraf and Wehgal, 2012; Eringen, 1966a):

∇.V=0, (1)

$$\rho_{f}\left(\frac{\partial V}{\partial t}+V.\nabla V\right)=-\nabla p+\left(\mu_{f}+k^{*}\right)\nabla^{2}V+k^{*}\left(\nabla \times v\right)+J\times B$$ (2)

$$\rho_{f}j\left(\frac{\partial v}{\partial t}+v.\nabla v\right)=\left(\alpha +\beta +\gamma \right)\nabla .\nabla v-\gamma \left(\nabla \times \nabla \times v\right)+k^{*}\left(\nabla \times v\right)-2kv,$$ (3)

Here, the velocity vector is represented by $V=\left(u,v,w\right)$ , pressure is indicated by p , microrotation is shown by v , and current density is indicated by $J=\sigma \left(V\times B\right)$ , with σ indicating electrical conductivity and B indicating the magnetic field. Moreover, the fluid density is represented by $\rho_{f}$ , dynamic viscosity is given by $\mu_{f}$ , vortex viscosity is given by $k^{*}$ , microinertia density is given by j , and gyroviscosity coefficients are given by α,β, and γ . It is noted that there are restrictions on $\alpha ,\beta ,\gamma ,\mu_{f},$ and $k^{*}$ .

$$k^{*}\geq 0,2\mu_{f}+k^{*}\geq 0,3\alpha +\beta +\gamma \geq 0,\left|\gamma \right|\geq \beta .$$

The constitutive equations for the micropolar fluids are described in greater detail in Eringen (1966a). The equations for thermal energy and diffusion of nanoparticles, which are based on the Buongiorno model and obey the laws of Fourier and Fick, are described in Abbas et al. (2019).

$$\rho_{f}c_{f}\left(\frac{\partial T}{\partial t}+V.\nabla T\right)=k_{f}\nabla^{2}T,$$ (4)

where the temperature is represented by T , specific heat of the nanofluid is given by $c_{f}$ , and thermal conductivity is given by $k_{f}$ .

Gyrotactic microorganisms are then taken into consideration according to Uddin et al. (2016).

$$\frac{\partial n}{\partial t}+\nabla J_{1}=0,$$ (5)

where

$$J_{1}=nV+n\tilde{V}-D_{n}\nabla_{n}$$

The flow of microorganisms caused by fluid convection is represented by $J_{1}$ , density of the gyrotactic microorganisms is indicated by n , and diffusivity of the microorganisms is given by $D_{n}$ . The cell swimming velocity is represented by the velocity vector $\tilde{V}=\left(0,0,\hat{w}\right)$ , where the velocity component along the z axis is represented by $\hat{w}=\left(b\;w_{c}\;/∆C\right)\nabla C$ . The maximal cell swimming speed is indicated by *w*_*c*_, and the chemotaxis constant is represented by *b*.

Using cylindrical coordinates, the governing Eqs. (1–6) (Hashmi et al., 2012; Ashraf and Wehgal, 2012) are simplified by taking into account a magnetic field of the form $B=\left(0,0,B\left(t\right)\right)$ , axisymmetric flow, and a microrotation vector normal to the disc surface $\left(v=\left(0,N,0\right)\right)$ .

### 2.3 Eyring–Powell fluid model

The Eyring–Powell model was introduced in 1994 for defining shear in a non-Newtonian flow. Here, the stress–strain relationship is shown using the non-Newtonian Eyring–Powell model by the strain–stress tensor Τ=ΡΙ+τ, where Ι denotes the identity stress tensor, Ρ signifies the pressure, and τ is the extra stress tensor satisfying the following relation (Rooman et al., 2022):

$$\tau =\mu \nabla V+\frac{1}{\beta_{1}}\sinh^{-1}\left(\frac{1}{c_{1}}\nabla V\right),$$ (6)

where

$$\sinh^{-1}\left(\frac{1}{c_{1}}\nabla V\right)\approx \left(\frac{1}{c_{1}}\nabla V\right)-\frac{1}{6}{\left(\frac{1}{c_{1}}\nabla V\right)}^{3},\left|\frac{1}{c_{1}}\nabla V\right|\ll 1$$

### 2.4 Governing equations and boundary conditions after applying assumptions

The Navier–Stokes flow is a form of fluid motion in which the typical dimension and flow rotation speed, $U_{w}$ , are both very small. Because a small Reynolds number is used to eliminate the inertial term in the Navier–Stokes equation, the Stokes estimate is frequently employed to explain the motion of magnetic microorganisms. Therefore, the fluid speed of the swimming magnetotactic bacteria is determined by the Navier–Stokes and continuity equations. The relevant governing equations to investigate the aforementioned fluid flow are as follows (Abbas et al., 2022; Shi et al., 2021):

$$\bar{r}\frac{\partial v}{\partial r}+r+R\frac{\partial u}{\partial s}=0,$$ (7)

$$\frac{u^{2}}{\bar{r}}-\frac{1}{\rho_{hnf}}\frac{\partial p}{\partial r}=0,$$ (8)

$$\begin{array}{ll} \left(\frac{\partial u}{\partial t}+\nu \frac{\partial u}{\partial r}+\frac{R}{\bar{r}}u\frac{\partial u}{\partial s}+u\nu \right) & =\\ & -\frac{1}{\rho_{hnf}}\frac{R}{\bar{r}}\frac{\partial p}{\partial s}+\frac{1}{\rho_{hnf}}\left(\left(\mu_{hnf}+k^{*}\right)+\frac{1}{\beta_{1}c_{1}}\right)\frac{\partial }{\partial r}\left(\frac{\partial u}{\partial r}+\frac{u}{\bar{r}}\right)\\ & -\frac{1}{6\beta_{1}{c_{1}}^{3}}\frac{\partial }{\partial r}{\left(\frac{\partial u}{\partial r}+\frac{u}{\bar{r}}\right)}^{3}\\ & -\frac{1}{\rho_{hnf}}\left(\sigma_{hnf}B^{2}\left(t\right)u-\frac{\mu_{hnf}}{k_{1}}u-k^{*}\frac{\partial N}{\partial r}\right)\frac{\partial N}{\partial t}+v\frac{\partial N}{\partial r}\\ & +\frac{Ru}{\bar{r}}\frac{\partial N}{\partial s}=-\frac{\gamma }{\rho_{hnf}j}\left(\frac{\partial^{2}N}{\partial r^{2}}+\frac{1}{\bar{r}}\frac{\partial N}{\partial r}\right)\\ & -\frac{k^{*}}{\rho_{hnf}j}\left(2N+\frac{\partial u}{\partial r}+\frac{u}{\bar{r}}\right) \end{array}$$ (9)

where *N* represents the microrotation velocity. Here, $\gamma^{*}=\left(\mu +\frac{k^{*}}{2}\right)=\mu \left(1+\frac{K_{1}}{2}\right)j,$ where $K_{1}=\frac{k^{*}}{\mu }$ represents the material parameter, $j=\frac{2vL}{ce^{\frac{s}{L}}}$ is the micro inertia per unit mass, and $\gamma^{*}$ and J indicate the spin gradient and vortex viscosity, respectively.

$${\left(\rho C_{p}\right)}_{hnf}\left(\frac{\partial T}{\partial t}+\nu \frac{\partial T}{\partial r}+\frac{R}{\bar{r}}u\frac{\partial T}{\partial s}\right)=\frac{1}{\bar{r}}\frac{\partial }{\partial r}\left(\bar{r}k_{hnf}\left(T\right)\frac{\partial T}{\partial r}\;\right)+\sigma_{hnf}B^{2}\left(t\right)u^{2},$$ (10)

$$\left(\frac{\partial \rho_{n}}{\partial t}+v\frac{\partial \rho_{n}}{\partial r}+\frac{R}{\bar{r}}u\frac{\partial \rho_{n}}{\partial s}\right)=D_{n}\left(\frac{\partial^{2}\rho_{n}}{\partial r^{2}}+\frac{1}{\bar{r}}\frac{\partial \rho_{n}}{\partial r}\right)+A\left(n,t\right)\rho_{n.}$$ (11)

Subject to the boundary conditions as per Ma et al. (2019), we get

$$\begin{array}{l} u=U_{w}=\frac{as}{1-ct},v=0,T=T_{w},\rho_{n}={\left(\rho_{n}\right)}_{w},N=-m\frac{\partial u}{\partial r},at\;r\to 0,\\ u\to 0,\frac{\partial u}{\partial r}\to 0,T\to T_{\infty },\rho_{n}\to {\left(\rho_{n}\right)}_{\infty },N\to 0,as\;r\to \infty \end{array}$$ (12)

Here, $\bar{r}=r+R$ . In the present discussion, it is assumed that $n>K_{m}$ , a>0 , and c≥0 with dimension ${\left(time\right)}^{-1}$ .

### 2.5 Similarity transformations and modelled ODEs

Consider the following dimensionless similarity transformation (Ma et al., 2019):

$$\begin{array}{l} \begin{array}{l} \eta =\sqrt{\frac{a}{\nu_{f}\left(1-ct\right)}}r,u=\frac{as}{1-ct}f^{\prime }\left(\eta \right),p=\rho_{f}{\left(\frac{as}{1-ct}\right)}^{2}P\left(\eta \right),\\ v=\frac{R}{\bar{r}}\sqrt{\frac{a\nu_{f}}{\left(1-ct\right)}}f\left(\eta \right),T=T_{\infty }+\left(T_{w}-T_{\infty }\right)\theta \left(\eta \right),N=\frac{as}{1-ct}\sqrt{\frac{a}{\nu_{f}\left(1-ct\right)}}g\left(\eta \right) \end{array}\\ \;\rho_{n}={\left(\rho_{n}\right)}_{\infty }+\left({\left(\rho_{n}\right)}_{w}-{\left(\rho_{n}\right)}_{\infty }\right)\chi \left(\eta \right),n=n_{\infty }+\left(n_{w}-n_{\infty }\right)\omega \left(\eta \right) \end{array}$$ (13)

The continuity equation is met by the dimensionless quantity in Eq. (13), and upon pressure elimination, the governing Eqs. (7–12) can be represented as follows:

$$\begin{array}{l} \left(\frac{\mu_{hnf}}{\mu_{f}}+K_{1}+\alpha_{1}\right)\left[f^{IV}+\frac{1}{\eta +K}\left(2f^{\prime \prime \prime }-\;\frac{f^{\prime \prime }}{\eta +K}+\frac{f^{\prime }}{{\left(\eta +K\right)}^{2}}\right)\right]\;-\;\left(\left(f^{\prime \prime }+\frac{f^{\prime }}{\eta +K}\right)\right)\left[\frac{\sigma_{hnf}}{\sigma_{f}}\;M+\frac{\mu_{hnf}}{\mu_{f}}\beta_{0}\right]\;-\\ \alpha_{2}\left[\begin{array}{l} \left({f^{\prime \prime }}^{2}+\frac{2f^{\prime }f^{\prime \prime }}{\eta +K}+\frac{{f^{\prime }}^{2}}{{\left(\eta +K\right)}^{2}}\right)f^{IV}+\left({f^{\prime \prime }}^{2}-\frac{3f^{\prime }f^{\prime \prime }}{\eta +K}-\frac{{f^{\prime }}^{2}}{{\left(\eta +K\right)}^{2}}\right)\frac{f^{\prime \prime }}{{\left(\eta +K\right)}^{2}}+\frac{3{f^{\prime }}^{3}}{{\left(\eta +K\right)}^{5}}\\ +2\left(f^{\prime \prime }+\frac{f^{\prime }}{\eta +K}\right){f^{\prime \prime \prime }}^{2}+2\left(3{f^{\prime \prime }}^{2}+\frac{2f^{\prime }f^{\prime \prime }}{\eta +K}-\frac{{f^{\prime }}^{2}}{{\left(\eta +K\right)}^{2}}\right)\frac{f^{\prime \prime \prime }}{\eta +K} \end{array}\right]-K_{1}\left(g^{\prime \prime }+\frac{g^{\prime }}{\eta +K}\right)+\\ \frac{\rho_{hnf}}{\rho_{f}}\;\left[\frac{K\;\left(f\;f^{\prime \prime \prime }-\;f^{\prime }\;f^{\prime \prime }\right)}{\eta +K}+\frac{K\;\left(f\;f^{\prime \prime }-\;{f^{\prime }}^{2}\right)}{{\left(\eta +K\right)}^{2}}-\;\frac{Kf\;f^{\prime }}{{\left(\eta +K\right)}^{3}}-\frac{\gamma }{\eta +K}\;\left(\frac{\eta }{2}\;f^{\prime \prime }+f^{\prime }\right)-\frac{\gamma }{2}\;\left(\eta f^{\prime \prime \prime }+3\;f^{\prime \prime }\right)\right]=0, \end{array}$$ (14)

$$\left(\frac{\mu_{hnf}}{\mu_{f}}+\frac{K_{1}}{2}\right)\left(g^{\prime \prime }+\frac{g^{\prime }}{\eta +K}\right)+\frac{K}{\eta +K}fg^{\prime }-\frac{K}{\eta +K}f^{\prime }g-K_{1}\left(2g+f^{\prime \prime }+\frac{f^{\prime }}{\eta +K}\;\right)-\frac{\gamma }{2}\left(\eta g^{\prime }+3g\right)=0,$$ (15)

$$\frac{k_{hnf}}{k_{f}\;}\;\left[\left(1+\beta \;\theta \right)\;\left(\theta^{\prime \prime }+\frac{\theta^{\prime }}{\eta +K}\right)+\beta \;{\left(\theta^{\prime }\right)}^{2}\right]+\frac{\sigma_{hnf}}{\sigma_{f}}MPrEc{f^{\prime }}^{2}+\frac{{\left(\rho C_{P}\right)}_{hnf}}{{\left(\rho C_{P}\right)}_{f}}\;\mathrm{Pr}\;\left[\left(\frac{Kf}{\eta +K}-\;\frac{\gamma \;\eta }{2}\right)\;\theta^{\prime }\right]=0,$$ (16)

$$\chi^{\prime \prime }+\frac{\chi }{\eta +K}+Lb\;\left[\left(\frac{Kf}{\eta +K}-\;\frac{\gamma \;\eta }{2}\right)\;\chi^{\prime }+\lambda \;\left(\Omega +\chi \right)\right]=0,$$ (17)

Similarly, pressure can be expressed as

$$\begin{array}{l} P\left(\eta \right)=\left(\frac{\mu_{hnf}}{\mu_{f}}+K_{1}+\alpha_{1}\right)\;\left[f^{\prime \prime \prime }+\frac{f^{\prime \prime }}{\eta +K}-\;\frac{f^{\prime }}{{\left(\eta +K\right)}^{2}}\right]+\frac{1}{2}\;\frac{\rho_{hnf}}{\rho_{f}}\left[f\;f^{\prime \prime }-\;{f^{\prime }}^{2}+\frac{f\;f^{\prime }}{\eta +K}-\frac{\eta +K}{K}\;\gamma \;\left(\frac{\eta }{2}\;f^{\prime \prime }+f^{\prime }\right)\right]\\ -\frac{\eta +K}{2K}\;\left(\frac{\sigma_{hnf}}{\sigma_{f}}M+\frac{\rho_{hnf}}{\rho_{f}}\beta_{0}\right)\;f^{\prime }+\alpha_{2}{\left(f^{\prime \prime }+\frac{f^{\prime }}{\eta +K}\right)}^{2}\left(f^{\prime \prime \prime }+\frac{f^{\prime \prime }}{\eta +K}-\;\frac{f^{\prime }}{{\left(\eta +K\right)}^{2}}\right), \end{array}$$ (18)

Subject to boundary conditions

$$\begin{array}{l} f^{\prime }\;\left(0\right)=\theta \;\left(0\right)=\chi \;\left(0\right)=\omega \;\left(0\right)=1,-m_{0}f^{\prime \prime }\;\left(0\right)=0,g\left(0\right)=0,=0,\\ f^{\prime }\;\left(\infty \right)=f^{\prime \prime }\;\left(\infty \right)=\theta \;\left(\infty \right)=\chi \;\left(\infty \right)=\omega \;\left(\infty \right)=0,g\left(\infty \right)=0. \end{array}$$ (19)

### 2.6 Thermophysical characteristics of the NF

#### 2.6.1 Hybrid nanomaterial properties

**FIGURE 2:**
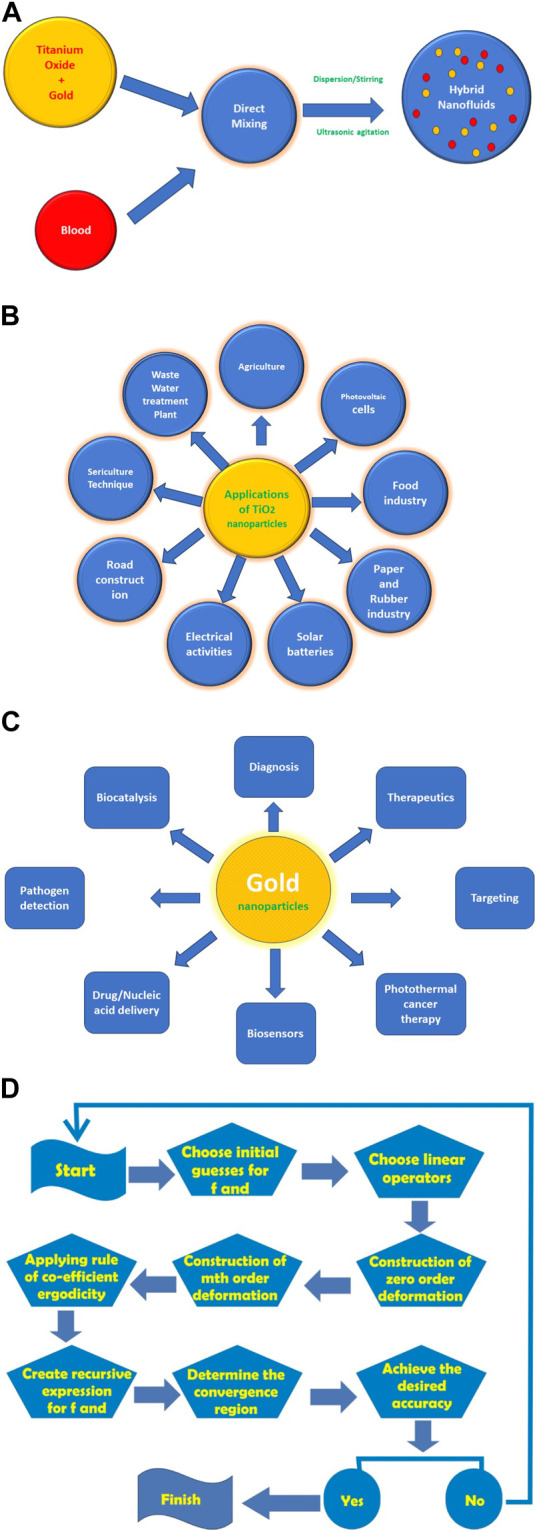
**(A)** Schematic for preparation of a hybrid nanofluid. **(B)** Schematic for applications of titanium dioxide ( $TiO_{2}$ ). **(C)** Schematic for applications of gold nanoparticles. **(D)** Flow chart for Homotopy Analysis Method.

The thermophysical characteristics and relations of the nano and hybrid nano materials are shown in Table 1 and Table 2, respectively. The properties and applications of the considered hybrid nanomaterials are shown in Figures 2A–C.

**TABLE 1:** Previous studies (Gul et al., 2015; Saqib et al., 2018; Ma et al., 2019) have analysed and reported the thermophysical characteristics of both the base fluid and HNF in great detail $\Phi_{1}+\Phi_{2}=\Phi$

Properties constituents	$c_{p}\left(\;J/\text{kg}∙K\right)$	$k\left(\;W/m∙K\right)$	$\sigma {\left(\Omega m\right)}^{-1}$	$\rho \left(\text{kg}/m^{3}\;\right)$
Gold ( Au )	129	318	$4.1\times 10^{6}$	19300
Titanium dioxide (TiO_2_)	4,250	8.9538	6.27 × 10^−5^	686.20
Blood	3594	0.492	$6.67\times 10^{-1}$	1063

**TABLE 2:** Thermophysical interactions of nano and hybrid nano fluids (Takabi and Salehi, 2014).

Properties	Nano and hybrid
Viscosity	$\frac{\mu_{hnf}}{\mu_{bf}}=\frac{1}{{\left(1-\phi_{g}-\phi_{TiO2}\right)}^{2.5}}$
Density	$\frac{\rho_{hnf}}{\rho_{bf}}=\phi_{g}\left(\frac{\rho_{g}}{\rho_{bf}}\right)+\phi_{TiO2}\left(\frac{\rho_{TiO2}}{\rho_{bf}}\right)+\left(1-\phi_{g}-\phi_{TiO2}\right)$
Thermal capacity	$\frac{{\left(\rho c_{p}\right)}_{hnf}}{{\left(\rho c_{p}\right)}_{bf}}=\phi_{g}\;\left(\frac{{\left(\rho c_{p}\right)}_{g}}{{\left(\rho c_{p}\right)}_{bf}}\right)+\phi_{TiO2}\left(\frac{{\left(\rho c_{p}\right)}_{g}}{{\left(\rho c_{p}\right)}_{bf}}\right)+\left(1-\phi_{g}-\phi_{TiO2}\right)$
Thermal conductivity	$\frac{k_{hnf}}{k_{bf}}=\frac{\frac{\left(\phi_{g}k_{g}+\phi_{TiO2}k_{TiO2}\right)}{\phi_{g}{+\phi }_{TiO2}}+\left\{\begin{array}{c} 2k_{bf}+2\left(\phi_{g}k_{g}+\phi_{TiO2}k_{TiO2}\right)\\ -2\left(\phi_{g}{+\phi }_{TiO2}\right)k_{bf} \end{array}\right\}}{\frac{\left(\phi_{g}k_{g}+\phi_{TiO2}k_{TiO2}\right)}{\phi_{g}{+\phi }_{TiO2}}+\left\{\begin{array}{c} 2k_{bf}-\left(\phi_{g}k_{g}+\phi_{TiO2}k_{TiO2}\right)\\ +\left(\phi_{g}{+\phi }_{TiO2}\right)k_{bf} \end{array}\right\}}$
Electrical conductivity	$\frac{\sigma_{hnf}}{\sigma_{bf}}=\frac{\frac{\left(\phi_{g}\sigma_{g}+\phi_{TiO2}\sigma_{TiO2}\right)}{\phi_{g}{+\phi }_{TiO2}}+\left\{\begin{array}{c} 2\sigma_{bf}+2\left(\phi_{g}\sigma_{g}+\phi_{TiO2}\sigma_{TiO2}\right)\\ -2\left(\phi_{g}{+\phi }_{TiO2}\right)\sigma_{bf} \end{array}\right\}}{\frac{\left(\phi_{g}k_{g}+\phi_{TiO2}k_{TiO2}\right)}{\phi_{g}{+\phi }_{TiO2}}+\left\{\begin{array}{c} 2\sigma_{bf}-\left(\phi_{g}\sigma_{g}+\phi_{TiO2}\sigma_{TiO2}\right)\\ +\left(\phi_{g}{+\phi }_{TiO2}\right)\sigma_{bf} \end{array}\right\}}$

**Table:** 

Notations and parameters in the governing equations			
Name of parameter	Symbol and formula	Name of parameter	Symbol and formula
Curvature factor	$K=\sqrt{\frac{a}{\nu_{f}\left(1-ct\right)}}R$	Eyring–Powell fluid model parameter	$\alpha_{2}=\frac{a^{3}s^{2}}{\beta_{1}c_{1}^{3}\rho_{f}\nu_{f}^{2}{\left(1-ct\right)}^{3}}$
Variable nutrient bacterial growth rate	$\left(n,t\right)=a\lambda \left(t\right)\frac{n}{\left(K_{m}+n\right)}$	Variable thermal conductivity	$\beta =\sqrt{\frac{\pi^{2}v_{f}}{a^{2}c}}$
Magnetic parameter	$M=\frac{\sigma_{f}B_{0}^{2}}{\rho_{f}a}$	Porosity parameter	$\beta_{0}=\frac{\mu_{f}\left(1-ct\right)}{\rho_{f}k_{1}a}$
Conversion factor	$Y=\frac{{\left(\rho_{n}\right)}_{w}-{\left(\rho_{n}\right)}_{\infty }}{n_{w}-n_{\infty }}$	Bioconvection Lewis number	$Lb=\nu_{f}/D_{n}$
Bacterial difference density parameter	$\Omega =\frac{{\left(\rho_{n}\right)}_{w}}{{\left(\rho_{n}\right)}_{w}-{\left(\rho_{n}\right)}_{\infty }}$	Non-dimensional generation/absorption coefficient	$Q=\frac{1-ct}{a{\left(\rho C_{P}\right)}_{f}}Q^{*}$
Maximum growth rate	$\left(t\right)=\frac{\lambda_{0}}{1-ct}$		
Eyring–Powell fluid model parameter	$\alpha_{1}=\frac{\mu_{f}}{\beta_{1}c_{1}}$	Prandtl number	$\mathrm{Pr}=\frac{\nu {\left(\rho C_{P}\right)}_{f}}{k_{0}}$ $\left(\mathrm{Pr}\approx 21\;for\;blood\right)$

#### 2.6.2 Physical quantities of relevance

Examining the behaviour of the local skin-friction coefficient ( $C_{fs}$ ) is worthwhile here, along with the Nusselt number ( ${Nu}_{s}$ ) and nutrient concentration number (Nn_*x*_). With respect to distinct physical aspects of the Nusselt and nutrient concentration number outcomes, the nondimensional local skin-friction coefficient are as follows:

$$C_{fs}=\frac{\tau_{w}}{\rho_{f}U_{w\;}^{2}},\;{Nu}_{s}=\frac{sq_{w}}{k_{f}\left(T_{w}-T_{\infty }\right)},\;{Nn}_{s}=\frac{sq_{n}}{D_{n}\left(n_{w}-n_{\infty }\right)}$$ (20)

where ${\;\tau }_{w}$ , ${\;q}_{w}$ , and $q_{n}$ are the wall surface shear stress, heat flux, and wall nutrient flux concentration, respectively, as given below:

$$\tau_{rs}={\left[\left(\mu_{nf}+k^{*}+\frac{1}{\beta c}\right)\left(\frac{\partial u}{\partial r}+\frac{u}{\bar{r}}\right)-\frac{1}{6\beta c^{3}}{\left(\frac{\partial u}{\partial r}+\frac{u}{\bar{r}}\right)}^{3}\right]}_{r=R},q_{w}=-k_{nf}{\left[\frac{\partial T}{\partial r}\right]}_{r=0},q_{s}=-D_{n}$$ (21)

These values can be expressed in nondimensional forms as. From Eq. (13), the non-dimensional equation related to skin friction as well as the heat transfer and nutrient concentration are finally determined as follows:

$$\begin{array}{c} C_{f}{\left(Re_{s}\right)}^{1/2}=\left(\varphi_{1}+\alpha_{1}\right)(f^{\prime \prime }\left(0\right)+\frac{f^{\prime }\left(0\right)}{\bar{\xi }})-\alpha_{2}{(f^{\prime \prime }\left(0\right)+\frac{f^{\prime }\left(0\right)}{\bar{\xi }})}^{3}\\ Nu_{s}{\left(Re_{s}\right)}^{-1/2}=-\theta^{/}\;\left(0\right)\\ Nn_{s}{\left(Re_{s}\right)}^{-1/2}=-\omega^{/}\;\left(0\right) \end{array}$$ (22)

where ${\left(Re_{s}\right)}^{1/2}=\sqrt{\frac{a}{\nu_{f}\left(1-ct\right)}}s$ is the local Reynolds number.

## 3 Solution methodology

To solve Eqs. (14–18) under the boundary constraints of Eq. (19), we employ the homotopy analysis method (HAM) with the following steps. Figure 2D shows the flow chart for this method.

The solutions having the auxiliary parameters ℏ adjust and control the convergence of the results.

The initial guesses are selected as follows:

$$f_{0}\left(\eta \right)=1-e^{-\eta },g_{0}\left(\eta \right)=e^{-\eta },\theta_{0}\left(\eta \right)=e^{-\eta },\chi_{0}\left(\eta \right)=e^{-\eta }.$$ (23)

The linear operators are taken as $L_{f},L_{g},L_{\theta },\text{and }L_{\chi }$ :

$$L_{f}\left(f\right)=f^{\prime \prime }-f^{\prime },L_{g}\left(g\right)=g^{\prime \prime }-g,L_{\theta }\left(\theta \right)=\theta^{\prime \prime }\;-\theta ,L_{\chi }\left(\chi \right)=\chi^{\prime \prime }-\chi ,$$ (24)

with the following properties:

$$L_{f}\left(c_{1}+c_{2}e^{-\eta }+c_{3}e^{\eta }\right)=0,L_{g}\left(c_{4}e^{-\eta }+c_{5}e^{\eta }\right)=0,L_{\theta }\left(c_{6}e^{\eta }+c_{7}e^{-\eta }\right)=0,L_{\chi }\left(c_{8}e^{-\eta }+c_{9}e^{\eta }\right)=0,$$ (25)

where $c_{i}\left(i=1-9\right)$ are the constants in the general solution.

## 4 Validation of the results

The outcomes of the validations are presented both numerically and graphically in this work. The numerical (ND-Solved) approaches are juxtaposed with the semi-analytical HAM results for the velocity distributions $f^{\prime }\left(\eta \right),g\left(\eta \right)$ , temperature profile $\theta \left(\eta \right)$ , and bacterial density field $\chi \left(\eta \right).$

**FIGURE 3:**
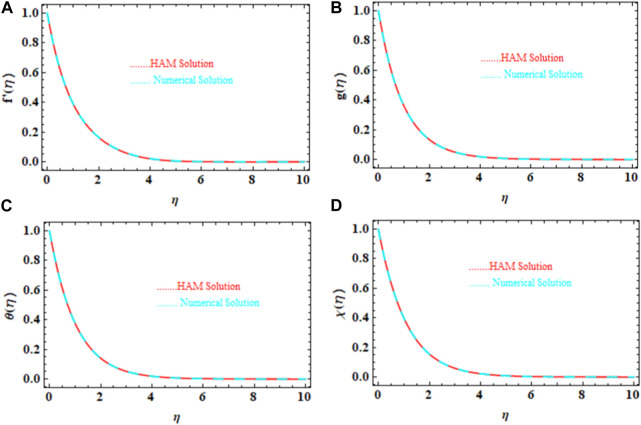
**(A–D)** Graphical validations of the HAM with numerical methods for $f^{\prime }\left(\eta \right),g\left(\eta \right),\theta \left(\eta \right)$ , and $\chi \left(\eta \right)$ .

Figures 3A–D display the comparisons of the HAM and numerical solutions for $f^{\prime }\left(\eta \right),g\left(\eta \right),\theta \left(\eta \right)$ , and $\chi \left(\eta \right)$ . For every profile, there is outstanding agreement between both outcomes. The HAM solution outcomes, numerical solution results, and absolute errors between $f^{\prime }\left(\eta \right),g\left(\eta \right)$ , $\theta \left(\eta \right),$ and $\chi \left(\eta \right)$ are presented in Tables 3–6. Between the two sets of results, it is noted that there is very good agreement for each profile.

**TABLE 3:** HAM and numerical technique (ND-Solve) validations for $\mathrm{f\prime}\left(\eta \right).$

$\prime \prime \eta^{\prime \prime }$	HAM solution	Numerical solution	Absolute error
0	$\prime \prime 1.000000^{\prime \prime }$	$\prime \prime 1.000000^{\prime \prime }$	$\prime \prime 0.000000^{\prime \prime }$
1	$\prime \prime 0.391988^{\prime \prime }$	$\prime \prime 0.392072^{\prime \prime }$	$\prime \prime 0.001674^{\prime \prime }$
2	$\prime \prime 0.163974^{\prime \prime }$	$\prime \prime 0.164194^{\prime \prime }$	$\prime \prime 0.004408^{\prime \prime }$
3	$\prime \prime 0.063938^{\prime \prime }$	$\prime \prime 0.064225^{\prime \prime }$	$\prime \prime 0.005738^{\prime \prime }$
4	$\prime \prime 0.020856^{\prime \prime }$	$\prime \prime 0.021091^{\prime \prime }$	$\prime \prime 0.004685^{\prime \prime }$
5	$\prime \prime 0.005242^{\prime \prime }$	$\prime \prime 0.005385^{\prime \prime }$	$\prime \prime 0.002868^{\prime \prime }$
6	$\prime \prime 0.000760^{\prime \prime }$	$\prime \prime 0.000833^{\prime \prime }$	$\prime \prime 0.001462^{\prime \prime }$
7	$\prime \prime -0.000148^{\prime \prime }$	$\prime \prime -0.000115^{\prime \prime }$	$\prime \prime 0.000662^{\prime \prime }$
8	$\prime \prime -0.000187^{\prime \prime }$	$\prime \prime -0.000173^{\prime \prime }$	$\prime \prime 0.000277^{\prime \prime }$
9	$\prime \prime -0.000106^{\prime \prime }$	$\prime \prime -0.000100^{\prime \prime }$	$\prime \prime 0.000111^{\prime \prime }$
10	$\prime \prime -0.000048^{\prime \prime }$	$\prime \prime -0.000046^{\prime \prime }$	$\prime \prime 0.000043^{\prime \prime }$

**TABLE 4:** HAM and numerical technique (ND-Solve) validations for $g\left(\eta \right).$

$\prime \prime \eta^{\prime \prime }$	HAM solution	Numerical solution	Absolute error
0	$\prime \prime 1.000000^{\prime \prime }$	$\prime \prime 1.000000^{\prime \prime }$	$\prime \prime 8.881780^{\prime \prime }\times 10^{-16}$
1	$\prime \prime 0.360849^{\prime \prime }$	$\prime \prime 0.360848^{\prime \prime }$	$\prime \prime 0.000459^{\prime \prime }$
2	$\prime \prime 0.133882^{\prime \prime }$	$\prime \prime 0.133881^{\prime \prime }$	$\prime \prime 0.000503^{\prime \prime }$
3	$\prime \prime 0.133882^{\prime \prime }$	$\prime \prime 0.049807^{\prime \prime }$	$\prime \prime 0.000269^{\prime \prime }$
4	$\prime \prime 0.018467^{\prime \prime }$	$\prime \prime 0.018467^{\prime \prime }$	$\prime \prime 0.000092^{\prime \prime }$
5	$\prime \prime 0.006823^{\prime \prime }$	$\prime \prime 0.006823^{\prime \prime }$	$\prime \prime 0.000024^{\prime \prime }$
6	$\prime \prime 0.002516^{\prime \prime }$	$\prime \prime 0.002516^{\prime \prime }$	$\prime \prime 5.105970^{\prime \prime }\times 10^{-6}$
7	$\prime \prime 0.000926^{\prime \prime }$	$\prime \prime 0.000926^{\prime \prime }$	$\prime \prime 9.825230^{\prime \prime }\times 10^{-7}$
8	$\prime \prime 9.825230^{\prime \prime }\times 10^{-7}$	$\prime \prime 0.000341^{\prime \prime }$	$\prime \prime 1.794910^{\prime \prime }\times 10^{-7}$
9	$\prime \prime 0.000125^{\prime \prime }$	$\prime \prime 0.000125^{\prime \prime }$	$\prime \prime 3.321050^{\prime \prime }\times 10^{-8}$
10	$\prime \prime 0.000046^{\prime \prime }$	$\prime \prime 0.000046^{\prime \prime }$	$\prime \prime 6.731680^{\prime \prime }\times 10^{-9}$

**TABLE 5:** HAM and numerical technique (ND-Solve) validations for $\theta \left(\eta \right).$

$\prime \prime \eta^{\prime \prime }$	HAM solution	Numerical solution	Absolute error
0	$\prime \prime 1.000000^{\prime \prime }$	$\prime \prime 1.000000^{\prime \prime }$	$\prime \prime 8.881780^{\prime \prime }\times 10^{-16}$
1	$\prime \prime 0.360849^{\prime \prime }$	$\prime \prime 0.360848^{\prime \prime }$	$\prime \prime 0.000459^{\prime \prime }$
2	$\prime \prime 0.133882^{\prime \prime }$	$\prime \prime 0.133881^{\prime \prime }$	$\prime \prime 0.000503^{\prime \prime }$
3	$\prime \prime 0.133882^{\prime \prime }$	$\prime \prime 0.049807^{\prime \prime }$	$\prime \prime 0.000269^{\prime \prime }$
4	$\prime \prime 0.018467^{\prime \prime }$	$\prime \prime 0.018467^{\prime \prime }$	$\prime \prime 0.000092^{\prime \prime }$
5	$\prime \prime 0.006823^{\prime \prime }$	$\prime \prime 0.006823^{\prime \prime }$	$\prime \prime 0.000024^{\prime \prime }$
6	$\prime \prime 0.002516^{\prime \prime }$	$\prime \prime 0.002516^{\prime \prime }$	$\prime \prime 5.105970^{\prime \prime }\times 10^{-6}$
7	$\prime \prime 0.000926^{\prime \prime }$	$\prime \prime 0.000926^{\prime \prime }$	$\prime \prime 9.825230^{\prime \prime }\times 10^{-7}$
8	$\prime \prime 9.825230^{\prime \prime }\times 10^{-7}$	$\prime \prime 0.000341^{\prime \prime }$	$\prime \prime 1.794910^{\prime \prime }\times 10^{-7}$
9	$\prime \prime 0.000125^{\prime \prime }$	$\prime \prime 0.000125^{\prime \prime }$	$\prime \prime 3.321050^{\prime \prime }\times 10^{-8}$
10	$\prime \prime 0.000046^{\prime \prime }$	$\prime \prime 0.000046^{\prime \prime }$	$\prime \prime 6.731680^{\prime \prime }\times 10^{-9}$

**TABLE 6:** : HAM and numerical technique (ND-Solve) validations for $\chi \left(\eta \right).$

$\prime \prime \eta^{\prime \prime }$	HAM solution	Numerical solution	Absolute error
0	$\prime \prime 1.000000^{\prime \prime }$	$\prime \prime 1.000000^{\prime \prime }$	$\prime \prime 1.110220^{\prime \prime }\times 10^{-16}$
1	$\prime \prime 0.398472^{\prime \prime }$	$\prime \prime 0.400921^{\prime \prime }$	$\prime \prime 0.002448^{\prime \prime }$
2	$\prime \prime 0.154709^{\prime \prime }$	$\prime \prime 0.156267^{\prime \prime }$	$\prime \prime 0.001558^{\prime \prime }$
3	$\prime \prime 0.059141^{\prime \prime }$	$\prime \prime 0.059895^{\prime \prime }$	$\prime \prime 0.000754^{\prime \prime }$
4	$\prime \prime 0.022469^{\prime \prime }$	$\prime \prime 0.022804^{\prime \prime }$	$\prime \prime 0.000335^{\prime \prime }$
5	$\prime \prime 0.008530^{\prime \prime }$	$\prime \prime 0.008675^{\prime \prime }$	$\prime \prime 0.000145^{\prime \prime }$
6	$\prime \prime 0.003245^{\prime \prime }$	$\prime \prime 0.003306^{\prime \prime }$	$\prime \prime 0.000062^{\prime \prime }$
7	$\prime \prime 0.001238^{\prime \prime }$	$\prime \prime 0.001264^{\prime \prime }$	$\prime \prime 0.000026^{\prime \prime }$
8	$\prime \prime 0.000474^{\prime \prime }$	$\prime \prime 0.000485^{\prime \prime }$	$\prime \prime 0.000011^{\prime \prime }$
9	$\prime \prime 0.000182^{\prime \prime }$	$\prime \prime 0.000186^{\prime \prime }$	$\prime \prime 4.690510^{\prime \prime }\times 10^{-6}$
10	$\prime \prime 0.000070^{\prime \prime }$	$\prime \prime 0.000072^{\prime \prime }$	$\prime \prime 1.971020^{\prime \prime }\times 10^{-6}$

## 5 Results and discussion

In this investigation, the HAM is used to evaluate the efficacy of several regulating elements, such as the volume fraction ϕ , curvature factor K , fluid parameter $\alpha_{1}$ , maximum growth rate of the bacteria λ , unsteady parameter γ , porosity parameter $\beta_{0}$ , magnetic parameter M , nondimensional bacterial density difference Ω , nondimensional generation/absorption coefficient, bioconvection Lewis number Lb , variable thermal conductivity β on the temperature $\theta \left(\xi \right)$ , velocity $f^{\prime }\left(\xi \right)$ , bacterial density field $\chi \left(\xi \right)$ , Nusselt number, skin friction, and density of nutrient concentration number, through graphs.

### 5.1 Velocity profile

**FIGURE 4:**
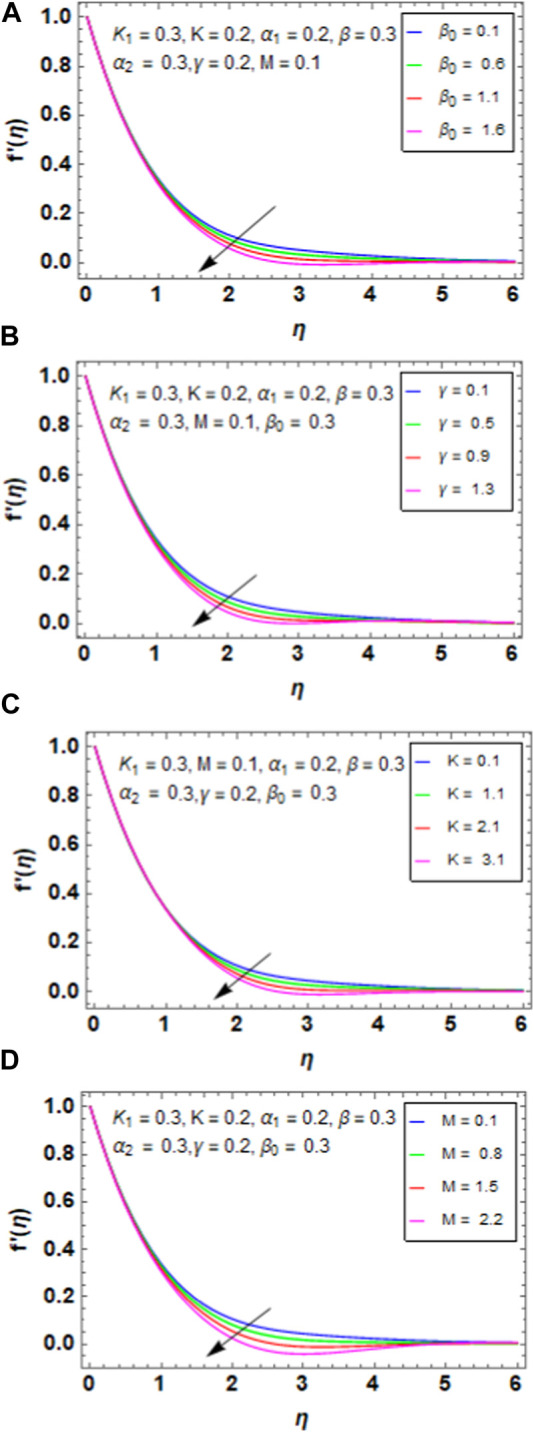
**(A)** Impact of $\beta_{0}$ on $f^{\prime }\left(\eta \right)$ . **(B)** Impact of γ on $f^{\prime }\left(\eta \right)$ . **(C)** Impact of K on $f^{\prime }\left(\eta \right)$ . **(D)** Impact of M on $f^{\prime }\left(\eta \right)$ .

Figure 4A shows the variations between the porosity parameter $\beta_{0}$ and velocity profile $f^{\prime }\left(\eta \right)$ . The porosity parameter is significant in various fields and disciplines owing to its influence on a wide range of physical, chemical, and engineering processes. When $\beta_{0}$ is enhanced, the velocity profile declines for the HNF; this is because a porous medium with increasing porosity usually has more empty areas. Although this may seem to improve flow at first glance, it also implies that less solid material is available to provide flow stability and order. A decrease in the fluid’s effective flow area therefore causes the velocity profile to diminish. Figure 4B explains the effect of the unsteady parameter γ on the velocity profile $f^{\prime }\left(\eta \right)$ . By enhancing the parameter γ , we can reduce the velocity profile $f^{\prime }\left(\eta \right)$ because an increase in γ indicates greater impacts of the time-dependent variables on the fluid flow. Increased fluid inertia property that tends to resist changes in velocity can result from this. The fluid may thus respond more slowly to changes in the external environment, causing the velocity profile to drop. Figure 4C represents the variation between the curvature factor K and velocity profile $f^{\prime }\left(\eta \right)$ . Increasing the curvature factor K reduces the velocity profile because the pressure gradient along a curved surface tends to get stronger as the curvature increases. A drop in the velocity profile may occur from this increased pressure gradient creating a greater barrier to flow. Because of higher pressure forces oppose the flow, the fluid has a tendency to slow down as it moves around the curved surface. Figure 4D represents the variation between the magnetic parameter M and velocity profile. As M increases, there is a decay in the velocity profile because the strength of the magnetic field operating on the NF increases with increase in M. The fluid’s magnetic nanoparticles prefer to align under this greater magnetic field, which might increase the effective viscosity of the NF. The velocity profile decreases as a result of the resistance of the increasing viscosity to flow.

### 5.2 Temperature profile

**FIGURE 5:**
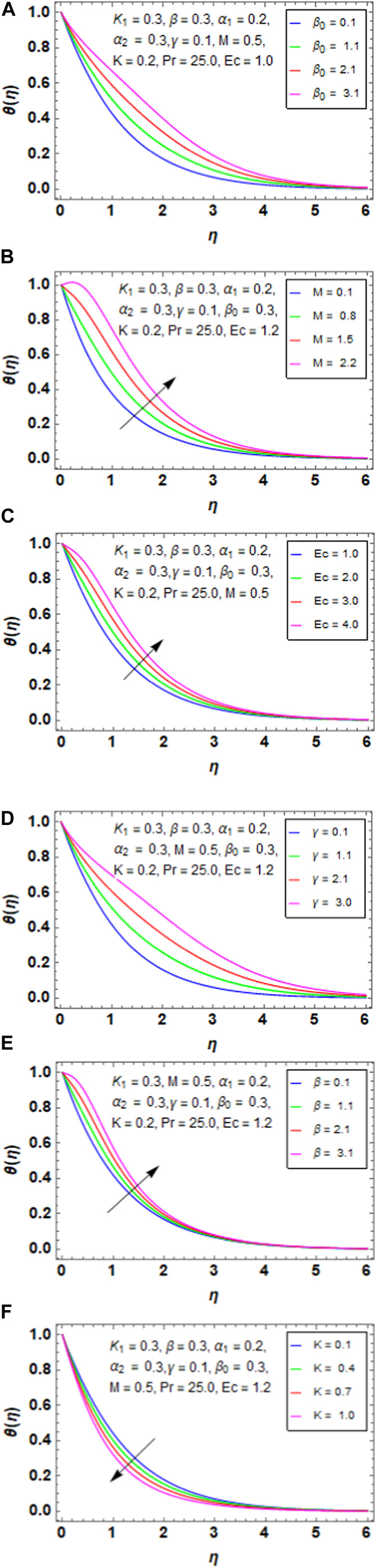
**(A)** Impact of $\beta_{0}$ on $\theta \left(\eta \right)$ . **(B)** Impact of M on $\theta \left(\eta \right)$ . **(C)** Impact of Ec on $\theta \left(\eta \right)$ . **(D)** Impact of γ on $\theta \left(\eta \right)$ . **(E)** Impact of β on $\theta \left(\eta \right)$ . **(F)** Impact of K on $\theta \left(\eta \right)$ .

Figure 5A presents the variation between the porosity parameter $\beta_{0}$ and temperature profile $\theta \left(\eta \right)$ . When $\beta_{0}$ increases, the temperature also increases because there is usually more space for flow within a porous medium when the porosity parameter increases. This may lead to improved convective heat transfer within the medium via increased fluid velocity and circulation. Figure 5B represents the variation between the magnetic parameter M and temperature profile $\theta \left(\eta \right).$ An increase in M increases the temperature profile of the HNF because the intensity of the external magnetic field acting on the nanoparticles increases with M . The fluid’s nanoparticles may move and align more significantly as a result of this increased magnetic force. Figure 5C represents the variation between the Eckert number Ec and temperature profile $\theta \left(\eta \right).$ As Ec increases, the temperature also increases because the kinetic energy contribution is greater than the enthalpy change when the Eckert number increases. This suggests that fluid motion, as opposed to heat transmission, accounts for a greater percentage of the system’s energy. More kinetic energy can improve fluid circulation and mixing, which can help the fluid transfer heat more effectively. Figure 5D presents the variation of the unsteady parameter γ with the temperature profile $\theta \left(\eta \right).$ As γ increases, the temperature also increases because the flow parameters will vary more quickly over time if the unsteady parameter γ is larger. Higher temperatures may be seen due to the greater heat dispersion and distribution across the fluid domain brought on by the enhanced fluid mixing. Figure 5E presents the variation between the variable thermal conductivity β and temperature profile $\theta \left(\eta \right).$ When we increase the variable thermal conductivity β , the temperature of the HNF increases because the variable thermal conductivity implies that the fluid’s capacity to transfer heat changes spatially. Better heat transmission throughout the fluid domain is made possible by the fluid’s greater ability to transmit heat when β increases. The fluid’s temperature may increase as a consequence of this increased heat conduction. Figure 5F presents the variation between the curvature factor K and temperature profile $\theta \left(\eta \right).$ When we increase the curvature factor K , the temperature of the HNF decreases because the surface area per unit volume often increases with increasing curvature; heat is dispersed from the fluid to the surroundings more efficiently owing to this larger surface area. As a result of improved cooling over the curved surface, the temperature of the HNF drops.

### 5.3 Microrotation profile

**FIGURE 6:**
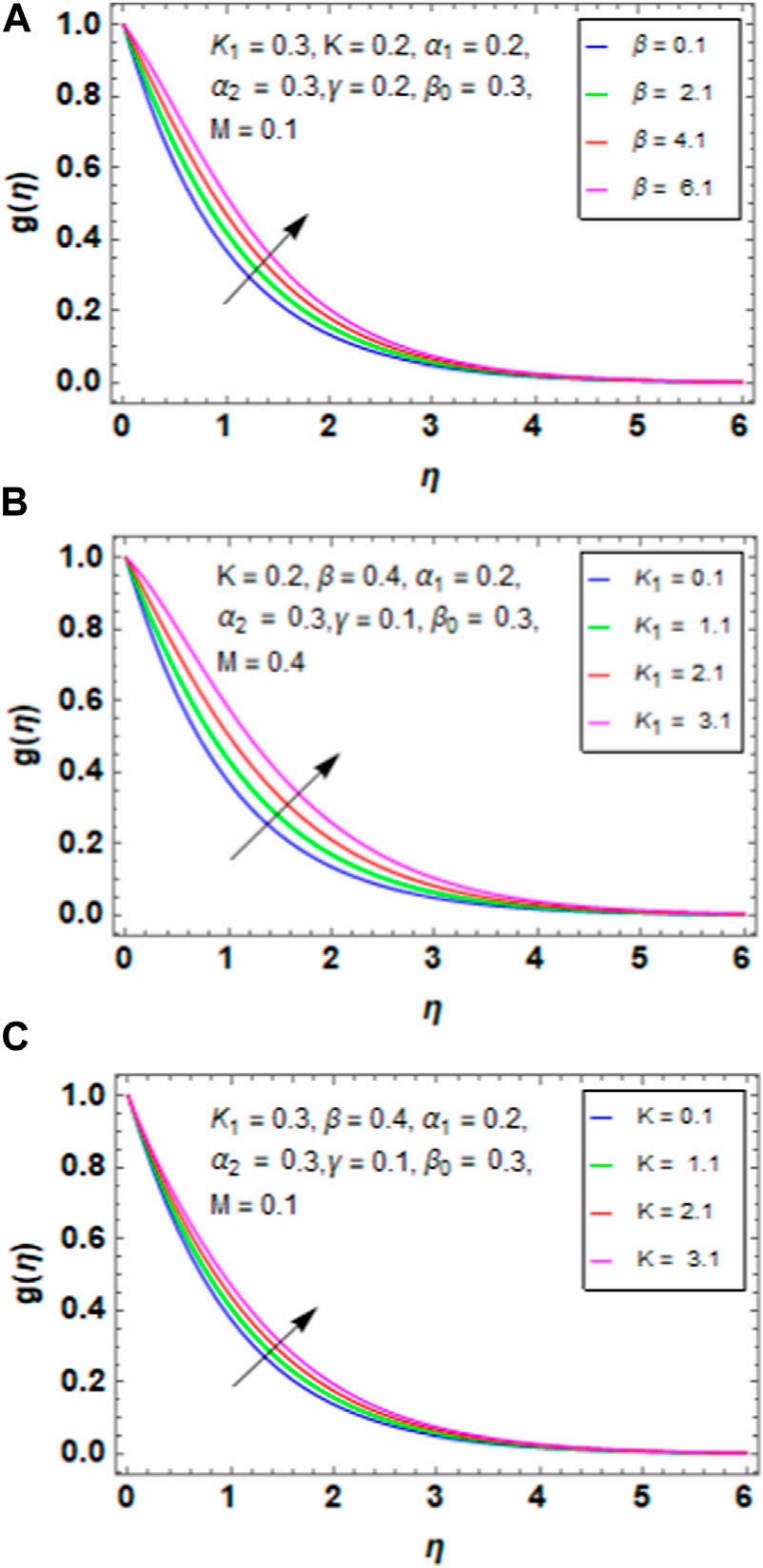
**(A)**Impact of β on $g\left(\eta \right)$ . **(B)** Impact of $K_{1}$ on $g\left(\eta \right)$ . **(C)** Impact of K on $g\left(\eta \right)$ .

Figure 6A presents the variation between the variable thermal conductivity β and micropolar profile $g\left(\eta \right).$ When we increase the variable thermal conductivity β , the micropolar profile of the HNF increases because a rise in β suggests that the thermal conductivity of the material is increasingly temperature-dependent. Higher temperatures thus lead to enhanced thermal conduction, which facilitates heat transmission within the fluid. The micropolar profile may increase as a result of this improved thermal conduction, enabling more effective temperature distribution throughout the fluid. Figure 6B presents the variation between the micropolar parameter $K_{1}$ and micropolar profile $g\left(\eta \right).$ When we increase the micropolar parameter $K_{1}$ , the micropolar profile of the HNF increases because the strength of the fluid’s microstructural effects, such as the microrotation and microdeformation of the fluid constituents, is represented by the micropolar parameter $K_{1}$ . These microstructural effects intensify as $K_{1}$ increases, thereby increasing the fluid’s degree of micropolar activity. The fluid’s total micropolar profile increases as a result of this enhanced micropolar behaviour. Figure 6C presents the variation between the curvature factor K and micropolar profile $g\left(\eta \right).$ When we increase the curvature factor K , the micropolar profile of the HNF increases because more complicated microstructural effects arise within the fluid as a result of the curved surface’s flow patterns being impacted by the increase in curvature K . These effects, which are more noticeable in areas with greater curvature, include microrotation and microdeformation of the fluid components. Consequently, the fluid’s micropolar activity intensifies, which increases the micropolar profile.

### 5.4 Bacterial density field

**FIGURE 7:**
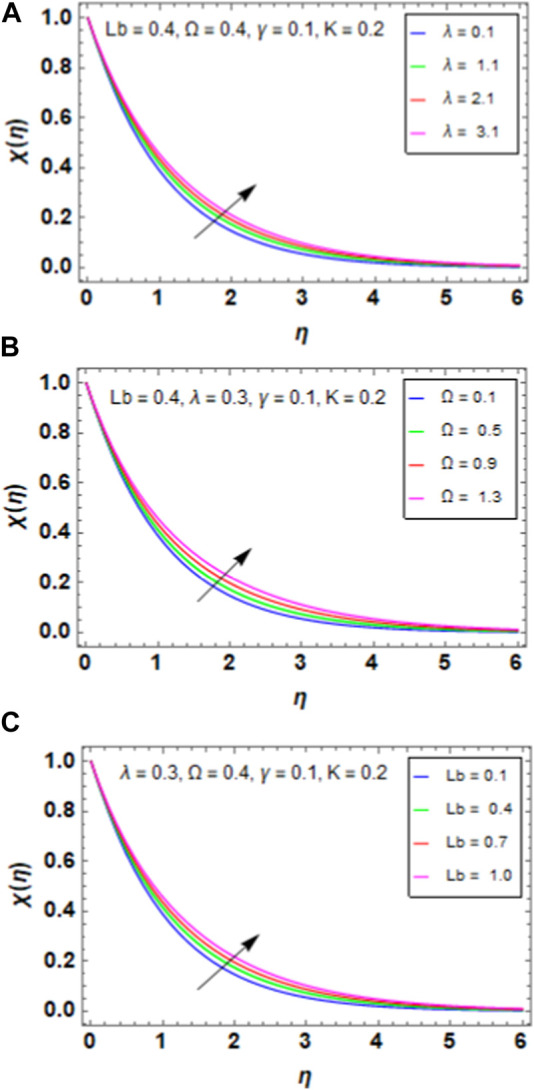
**(A)** Impact of λ on $\chi \left(\eta \right)$ . **(B)** Impact of Ω on $\chi \left(\eta \right)$ . **(C)** Impact of Lb on $\chi \left(\eta \right)$ .

Figure 7A presents the variation between the maximum growth rate of the bacteria λ and bacterial density field $\chi \left(\eta \right)$ . When we increase the maximum growth rate of the bacteria λ , the bacterial density field of the HNF increases because the bacteria that have a greater maximal growth rate are believed to multiply more quickly. Consequently, the fluid produces more bacteria in a given amount of time, increasing the total bacterial density field. Figure 7B presents the variation between the bacterial difference density parameter Ω and bacterial density field $\chi \left(\eta \right)$ . When we increase the bacterial difference density parameter Ω , the bacterial density field of the HNF increases because the density difference between the bacteria and surrounding fluid is represented by the bacterial differential density parameter Ω. This suggests that the bacteria become more buoyant in relation to the fluid as Ω increases. The bacteria multiply and spread more broadly in the fluid as a result of this enhanced buoyancy, increasing the total bacterial density field. Figure 7C presents the variation between the bioconvection Lewis number Lb and bacterial density field $\chi \left(\eta \right)$ . When we increase the bioconvection Lewis number Lb , the bacterial density field of the HNF increases because the transport of nutrients to the bacterial cells is more effective when Lb increases. The increase in total bacterial density field inside the HNF is a result of the increased nutrition availability, which encourages bacterial growth and replication.

### 5.5 Graphical and numerical results of the skin-friction coefficient, Nusselt number, and nutrient concentration number

**FIGURE 8:**
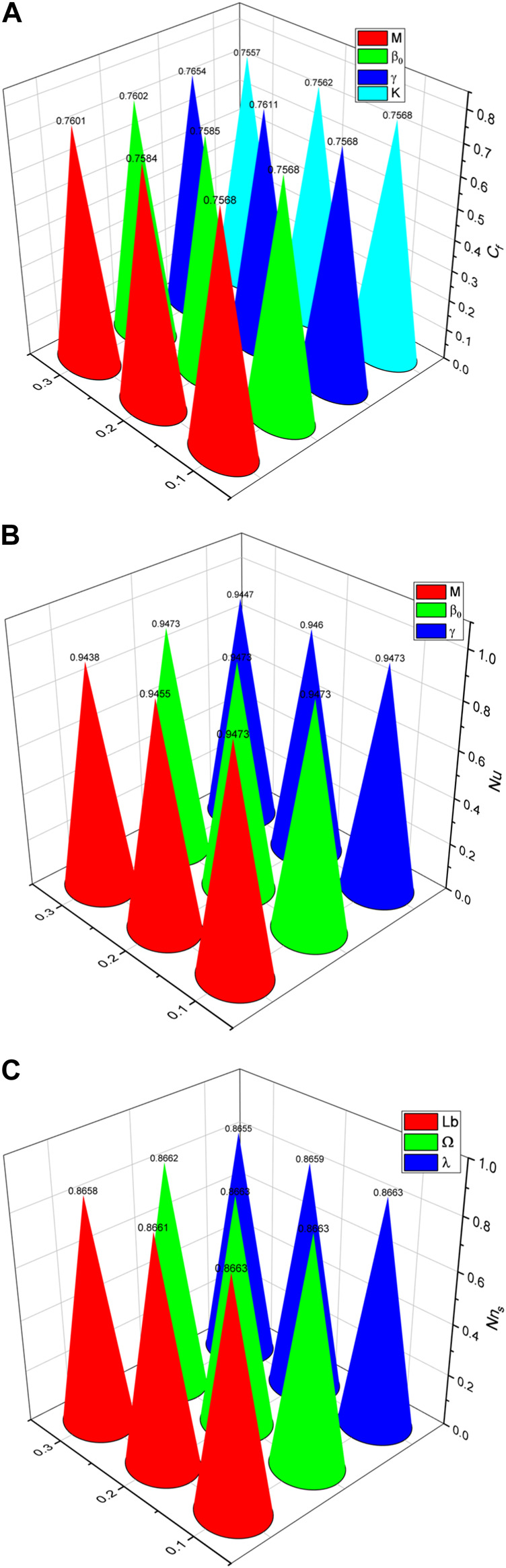
**(A)** Variations in skin friction $C_{f}{\left(Re_{s}\right)}^{1/2}$ for several values of $\gamma ,\beta_{0},M$ , and K. **(B)** Variations in the Nusselt number $Nu_{s}{\left(Re_{s}\right)}^{-1/2}$ for several values of $\beta_{0},M$ , and γ. **(C)** Variations in the nutrient concentration number $Nn_{s}{\left(Re_{s}\right)}^{-1/2}$ for several values of Lb,Ω , and λ. .

Figure 8A shows how the skin-friction coefficient is affected by η , $\beta_{0}$ , M , and K . Changes in the porous structure of the medium significantly affect the frictional forces, as demonstrated by the porosity parameter $\beta_{0}$ that has the greatest impact on skin friction. The magnetic parameter M has less of an impact on skin friction than the porosity parameter ${\;\beta }_{0}$ . This suggests that in terms of frictional forces, the magnetic field modifies flow behaviour to a lesser extent. Although not as much as the porosity parameter $\beta_{0}$ , the unsteady parameter γ also increases skin friction. This parameter shows that the frictional forces are influenced by temporal variations in the flow conditions. Out of all the characteristics examined, the curvature factor K has the least incremental impact on skin friction; this implies that the frictional forces are mostly determined by factors other than the surface geometric curvature, such as porosity, magnetic fields, and flow unsteadiness.

The changes in the Nusselt number $Nu_{s}{\left(Re_{s}\right)}^{-1/2}$ for different values of $\beta_{0}$ , M , and γ are shown in Figure 8B. Stronger magnetic fields appear to inhibit heat transport, as seen by the continuous decline in the Nusselt number as the magnetic parameter M increases. This behavior is consistent with the findings of other studies, which shows that the increased flow mixing brought about by higher magnetic fields can lower the heat transfer rate. The results therefore emphasise the necessity of considering magnetic fields carefully in heat transfer applications, particularly when aiming to improve or regulate the heat transfer rate. Heat transmission is impacted by flow unsteadiness, as seen by the decreasing Nusselt number as the unsteady parameter γ increases. Typically, unsteady flows show variations in temperature gradients and velocity, which may interfere with the formation of boundary layers and lower the effectiveness of heat transmission. As a result, engineering systems functioning in unstable environments could experience reduced heat transfer efficiency, requiring countermeasures to preserve the intended thermal properties. Further investigation is necessary to fully understand the role of porosity in heat transfer processes involving magnetic fields and unsteady flows, as evidenced by the lack of discernible variations in the Nusselt number with increasing values of the porosity parameter $\beta_{0}$ . This finding contrasts with the significant influences of the magnetic and unsteady parameters.

The fluctuations of the nutrient concentration number for various values of Lb,Ω , and λ are shown in Figure 8C. There are substantial associations between these variables, as shown by the correlations between the influencing factors and nutrient concentration density provided. A reduction in the density of the nutrient concentration is observed with increases in the bioconvection Lewis number Lb, bacterial density difference Ω , and bacterial maximum growth rate λ . These results have significant ramifications for medical cancer therapies that use artificial bacteria and magnetite nanoparticles. One may control the availability of the nutrients in normal cells and decrease tumour cell usage of such nutrients at the same time by adjusting the growth rate and density differential between the bacteria. Because the healthy cells are fed even as the tumour cells are starved, this focused strategy may increase the effectiveness of cancer therapies.

Table 7 illustrates the impacts of varying values of the magnetic parameter M , porosity parameter ${\;\beta }_{0}$ , unsteady parameter γ , and curvature factor K on skin friction. The findings indicate positive correlations between increasing values of these parameters and heightened skin friction. Notably, the porosity parameter ${\;\beta }_{0}$ exhibits the most pronounced enhancement in skin friction compared to the magnetic parameter M , unsteady parameter γ , and curvature factor K . Conversely, the least incremental effect on skin friction is observed with the curvature factor K as compared to the magnetic parameter M , unsteady parameter γ , and porosity parameter ${\;\beta }_{0}$ .

**TABLE 7:** Numerical values of skin friction $C_{f}{\left(Re_{s}\right)}^{1/2}$ for several values of $\gamma ,{\;\beta }_{0},M$ , and K.

M	$\beta_{0}$	γ	K	$C_{f}{\left(Re_{s}\right)}^{1/2}$
0.1	0.1	0.1	0.1	0.7568
0.2				0.7584
0.3				0.7601
	0.1			0.7568
	0.2			0.7585
	0.3			0.7602
		0.1		0.7568
		0.2		0.7611
		0.3		0.7654
			0.1	0.7568
			0.2	0.7562
			0.3	0.7557

Table 8 illustrates the trend of the Nusselt number for incremental values of the magnetic parameter M , porosity parameter ${\;\beta }_{0}$ , and unsteady parameter γ . The data indicate consistent decreases in the Nusselt number as the values of the magnetic parameter M and unsteady parameter γ increase. Conversely, there is no discernible variation observed with increasing values of the porosity parameter ${\;\beta }_{0}$ . This suggests that the magnetic parameter M and unsteady parameter γ exert significant influences on the Nusselt number, leading to a decline, while the porosity parameter ${\;\beta }_{0}$ remains relatively unaffected. Table 9 presents the correlation between the nutrient concentration density and three influential factors: bioconvection Lewis number Lb , bacterial density difference Ω, and maximum growth rate of the bacteria λ . The analysis reveals that increases in the bioconvection Lewis number, bacterial density difference, and maximum growth rate are correlated with decreases in the nutrient concentration density. These mathematical findings suggest careful regulation of the bacterial density difference and bacterial growth rate in medical cancer treatments involving magnetite nanoparticles and artificial bacteria. This regulation may enhance nutrient availability in the normal cells while reducing nutrient utilisation by the tumour cells. In the context of medical treatment, it is advisable to augment the applied magnetic factors while diminishing the ratio of thermal diffusivity to mass diffusivity; this approach aims to optimise the nutrient consumption in normal cells while minimising that in tumour cells. Table 10 shows comparisons for skin friction values with findings from previous work, and it is seen that these results are in good agreement.

**TABLE 8:** Numerical values of the Nusselt number $Nu_{s}{\left(Re_{s}\right)}^{-1/2}$ for several values of $\beta_{0},M,$ and γ.

M	$\beta_{0}$	γ	$Nu_{s}{\left(Re_{s}\right)}^{-1/2}$
0.1			0.9473
0.2			0.9455
0.3			0.9438
	0.1		0.9473
	0.2		0.9473
	0.3		0.9473
		0.1	0.9473
		0.2	0.9460
		0.3	0.9447

**TABLE 9:** Numerical values of the nutrient concentration number $Nn_{s}{\left(Re_{s}\right)}^{-1/2}$ for several values of Lb,Ω, and λ.

Lb	Ω	λ	$Nn_{s}{\left(Re_{s}\right)}^{-1/2}$
0.1			0.8663
0.2			0.8661
0.3			0.8658
	0.1		0.8663
	0.2		0.8663
	0.3		0.8662
		0.1	0.8663
		0.2	0.8659
		0.3	0.8655

**TABLE 10:** Comparison of skin friction values with literature.

M	Present results	Elgazery et al. (Elgazery et al., 2022)
1.0	1.4188276394073485	1.4142165596981353
5.0	2.431230962861769	2.4494934810118005
10.0	3.3163485207768746	3.3166680277801750
50.0	7.184288470017636	7.1414769000363100
100.0	10.038094657157158	10.049923999999939

## 6 Conclusion

A mathematical analysis of the impact of heat generation on an unsteady magnetised Powell–Eyring micropolar blood-based hybrid nanofluid over a curved surface is reported in this work. This model’s objective is to contrast the performances of the HNF models. The effects of thermal jump and velocity slip are considered when analysing the curved surface. The mathematical model was formulated based on the underlying flow assumptions. The cylindrical surface is utilised to calculate the flow quantities, and the outcomes are visually presented using graphs and tables. The following conclusions may be drawn from the results of this work:

• This study significantly contributes to literature by uncovering novel flow features that were previously unexplored.
• The utilisation of gold nanoparticles shows potential for enhancing blood circulation and presents a promising therapeutic strategy for combating arterial diseases, in contrast to copper and aluminium oxide nanoparticles.
• The proposed strategy has advantages for effective delivery of medication through blood, as seen from the graphical findings and numerical solutions.
• The ratio of fluid to surface increases and rate of heat transfer decreases when the magnetic field is increased.
• Heat transmission enhancement increases the Biot number value. It was found that the blood velocity profile could be improved by increasing the values of the unstable parameters.
• As the porosity parameter, magnetic parameter, and magnetite volume percentage increase, the velocity distribution decreases.
• The distribution of blood temperature increases with the concentration of the magnetite nanoparticles. It is therefore possible to enhance the physical properties of the blood by submerging the magnetite nanoparticles.
• The current findings show that boosting the heat transfer rate is dependent on the magnetic parameter and Eckert number.
• The increasing behaviour of skin friction (C_f_) with increasing values of the magnetic parameter (M) for the HNF is observed.
• The increase in the total bacterial density field inside the HNF is a result of the increased availability of nutrition, which encourages bacterial growth and replication.
• As the cooling over the curved surface improves, the temperature of the HNF drops.

## Figures and Tables

**Table:** 

**Symbols**	**Quantity**	**Units**
$\left(r,s\right)$	Curvilinear coordinates	‐
$\left(u,v\right)$	Velocity components	m/s
N	Microrotation velocity	m/s
p	Pressure	$N/m^{2}$
T	Temperature	K
R	Radius of curvature	m
k	Thermal conductivity	W/m∙K
$k_{1}$	Permeability constant	
n	Nutrient concentration	mg/L
$D_{n}$	Diffusivity of nutrient	‐
K	Curvature constraint	‐
Pr	Prandtl number	‐
Lb	Lewis number	‐
B	Magnetic field	T
Q	Coefficient of generation/absorption	‐
**Greek symbols**	
$\alpha_{1},\alpha_{2}$	Fluid parameters	$kg/m^{3}$
$\mu_{f}$	Dynamic viscosity	kg/m∙s
ν	Kinematic viscosity	$m^{2}/s$
ρ	Density	$kg/m^{3}$
β	Thermal conductivity parameter	W/m∙K
σ	Electrical conductivity	S/m
$k^{*}$	Vortex viscosity	‐
j	Microinertial density	‐
**Subscripts**	
hnf	Hybrid nanofluid	
f	Base fluid	
p	Nanoparticles	
w	At the curved surface	
∞	Far away from the surface (at infinity)	

## Data Availability

The data that support the findings of the study are available from the corresponding author upon reasonable request.
